# Astragalus Polysaccharides Alleviate Inflammatory Damage in Murine Rheumatoid Arthritis by Modulating Th17/Treg Balance via the Gut–Joint Axis

**DOI:** 10.3390/molecules31203788

**Published:** 2026-10-21

**Authors:** Pu Yao, Jing Huang, Wenying Liu, Qian Yuan, Jun Lv

**Affiliations:** 1 Department of Pharmacy, The First Affiliated Hospital (Southwest Hospital) of Third Military Medical University ( Army Medical University), Chongqing 400038, China; 2 Department of Vascular and Endovascular Surgery, The First Affiliated Hospital (Southwest Hospital) of Army Medical University, Chongqing 400038, China; 3 Department of Dermatology, The First Affiliated Hospital (Southwest Hospital) of Army Medical University, Chongqing 400038, China

**Keywords:** astragalus polysaccharide, natural bioactive polymer, rheumatoid arthritis, Th17/Treg, gut microbiota

## Abstract

Astragalus polysaccharides (APSs) have demonstrated protective effects in RA models; however, the underlying microbe–host crosstalk remains unclear. In this study, collagen-induced arthritis (CIA) was established in mice, followed by prophylactic and therapeutic APS administration. The following were then assessed: articular histopathology, splenic Th17/Treg frequencies via flow cytometry, serum and joint cytokine profiles via ELISA and immunohistochemistry, gut microbial metagenomes, and bone erosion via Safranin O–fast green staining. It was found that APS treatment significantly attenuated joint inflammation and bone destruction, rebalanced the Th17/Treg ratio, and reduced pro-inflammatory cytokines (TNF-α and IL-17) while elevating anti-inflammatory IL-10 both systemically and locally. Subsequently, fecal microbiota from APS-treated donor mice was transplanted via oral gavage into CIA recipient mice. APS reversed RA-associated gut dysbiosis by enriching *Ligilactobacillus*, *Lactobacillus*, *Limosilactobacillus*, and *Bacteroides*, and these microbial shifts closely paralleled diminished articular inflammation and preserved bone architecture. Collectively, these findings indicate that APS mitigates RA progression by reconfiguring the gut microbial ecosystem—favoring beneficial taxa while curbing pathobionts—thereby restoring intestinal homeostasis, normalizing the Th17/Treg axis, and suppressing systemic and articular inflammatory responses.

## 1. Introduction

Rheumatoid arthritis (RA) is an autoimmune and chronic inflammatory disease, with a global prevalence exceeding 0.5% [1]. It is now widely accepted that immune dysregulation is closely related to the development of RA. For example, the infiltration of myeloid and helper T cells into joints accelerates disease progression, although the specific mechanisms remain unclear [2]. Clinically, RA presents as symmetrical joint lesions, synovial inflammation, and local cartilage degeneration, commonly affecting the hands and feet, with patients exhibiting joint deformities, swelling, and pain [2]. Current clinical treatments primarily focus on suppressing inflammation and relieving pain, with limited therapeutic options, including biologics and glucocorticoids [3]. Although these drugs effectively alleviate some clinical symptoms, they do not fundamentally resolve the underlying immune dysregulation and increase the risk of complications such as hepatotoxicity and nephrotoxicity [4]. Therefore, it is necessary to identify a therapeutic agent that can fundamentally regulate autoimmune imbalance and restore homeostasis to improve the efficacy of RA treatment.

Astragalus polysaccharides (APSs) are water-soluble macromolecular active components extracted from Astragalus membranaceus, possessing immunomodulatory and antioxidant functions. APSs can regulate the immune activity of various immune cells, including T cells, B cells, and macrophages, and ameliorate immunosuppression induced by drugs in immune organs. APSs can directly activate macrophages and T cells to release inflammatory mediators, exerting pro- or anti-inflammatory effects in infections and diabetes. As drugs with significant immunomodulatory value, APSs have also demonstrated therapeutic efficacy in multiple autoimmune diseases. APSs alleviated collagen overaccumulation in bleomycin-induced scleroderma mice by inhibiting TGF-β1 expression in skin cells [5]. In experimental autoimmune encephalomyelitis (EAE) mice, APSs delayed the onset of symptoms by activating the AMPK/JAK/STAT3/arginase-1 signaling cascade to suppress CD4^+^ T cells [6]; it inhibited neurotoxicity by activating Sema4D-PlexinB2 signaling to suppress crosstalk between MIMS and AIMS [7]; and it regulated microglial polarization via miR-155 to reduce inflammatory mediator release, thereby inhibiting the activation of neurotoxic astrocytes.

In our previous study, a 20 kDa polysaccharide isolated from Astragalus membranaceus was shown to alleviate dextran sulfate sodium (DSS)-induced colitis in mice by suppressing NF-κB activity [8]. Recent studies have confirmed the therapeutic effects of APSs on RA. APSs effectively reduced inflammatory indices in adjuvant-induced arthritis mice, decreased intra-articular cell accumulation, and alleviated joint swelling [9]. APSs can also modulate PI3K/AKT/mTOR autophagy, promote apoptosis, inhibit inflammation, and slow RA progression [10]. Existing studies have confirmed that APSs can directly target and regulate CD4^+^ T cell subsets to improve autoimmune inflammation. However, emerging evidence has established the gut microbiota as a central upstream regulator of the peripheral Th17/Treg immune balance, which serves as a core pathological axis driving RA inflammatory progression. Intestinal microbiota dysbiosis is tightly correlated with aberrant Th17/Treg differentiation, excessive intestinal and systemic inflammation, and aggravated arthritic phenotypes. Although the direct immunoregulatory effects of APSs have been well documented, whether APSs can reshape gut microbial homeostasis to indirectly mediate Th17/Treg immune balance and exert anti-arthritic effects remains poorly elucidated, representing a critical unresolved mechanism underlying APSs-mediated RA treatment.

Helper T cells are important regulators in RA, yet the mechanism by which APSs influence RA progression through T cells regulation remains unclear. Expansion of Prevotella copri correlates with enhanced susceptibility to arthritis [11]; metagenome-wide analyses show that the oral and gut microbiomes are perturbed in RA and partly normalized after treatment [12]; and transfer of Prevotella-dominated faecal microbiota from RA patients into germ-free arthritis-prone mice activates autoreactive T cells, increases intestinal Th17 cells and aggravates arthritis [13]. This study investigated the Treg/Th17 ratio in the blood and gut microbial abundance in RA mice before and after APS treatment, analyzing the mechanism by which APSs control gut microbial homeostasis to regulate the differentiation efficiency of Th17 and Treg cells, thereby influencing RA disease progression.

## 2. Results

### 2.1. APS Effectively Attenuates Bone Destruction and Delays RA Progression

**Figure 1:**
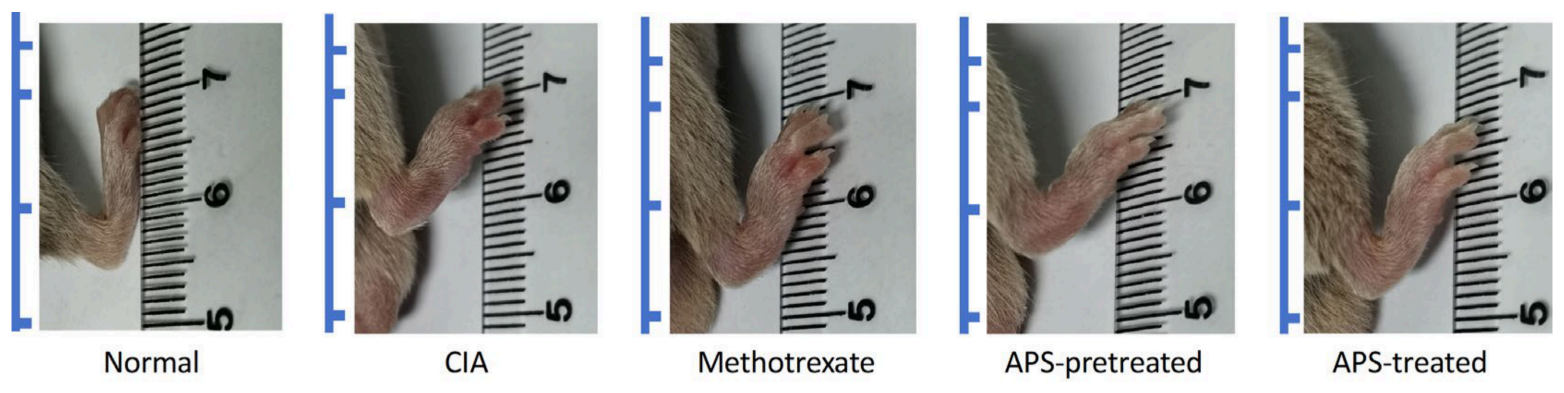
Morphological observation of the hind paws of mice in the normal, CIA, CIA + methotrexate, APS-pretreated, and APS-treated groups (*n* = 8).

**Figure 2:**
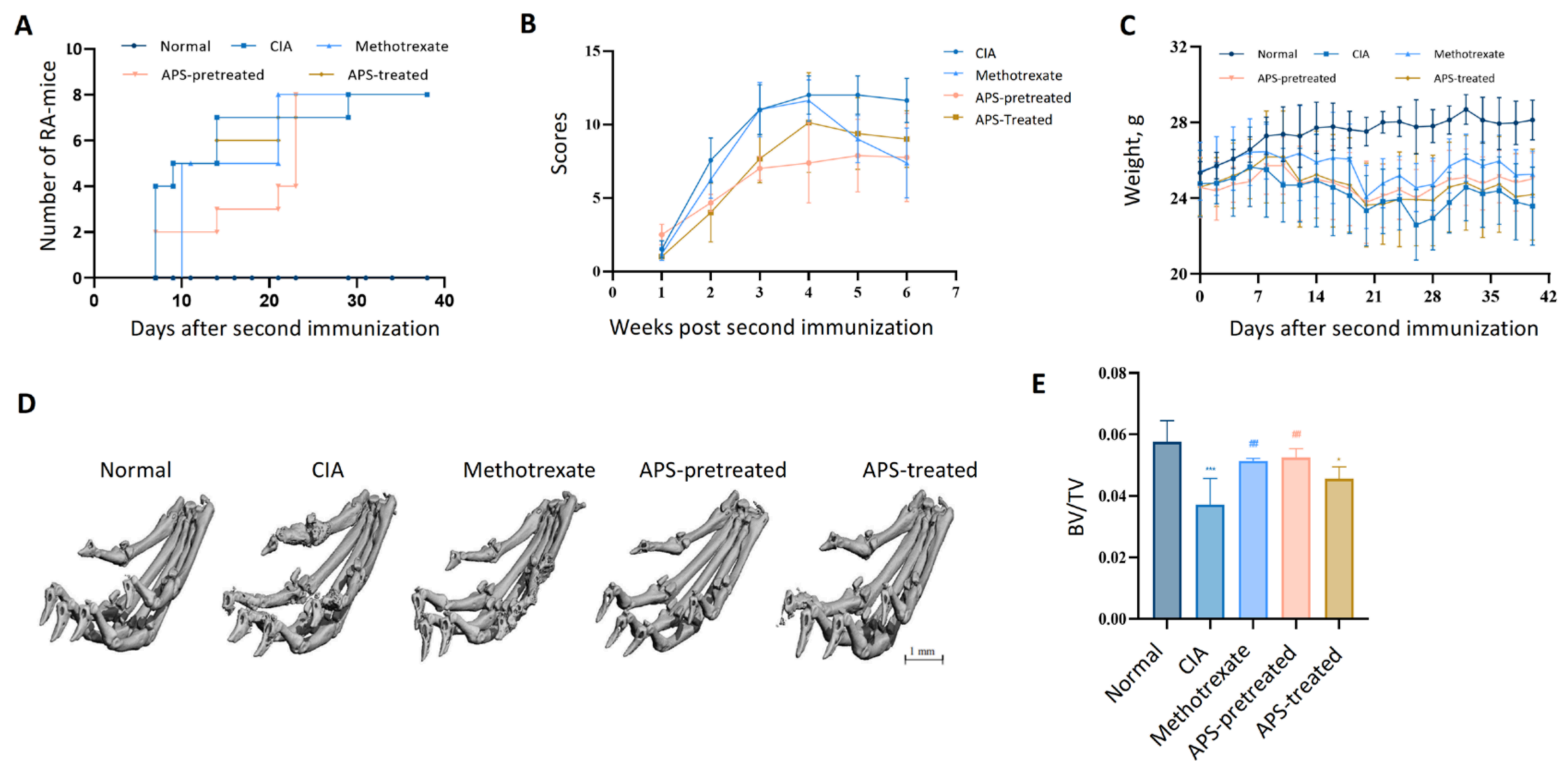
Detection of APS prevention and treatment of RA. (**A**) The number of RA symptoms in mice after the second immunization. (**B**) Assessment of the progression of arthritis symptoms in mice after the second immunization. (**C**) Assessment of the weight in mice after the second immunization *(n* = 8). CIA mouse arthritis symptom scoring criteria: 0 points—normal morphology, no erythema, and no joint redness or swelling; 1 point—single area involved, with ankle joint/metatarsophalangeal bone/palm erythema or mild swelling, or two areas involved, with erythema/swelling extending to the middle of the foot; 3 points—extensive involvement of the middle of the foot and erythema + moderate swelling of the ankle joint; 4 points—overall foot involvement, moderate redness, and swelling or joint stiffness/deformity/ulcers on the entire foot (including ankle, foot, and toe). (**D**) Micro-CT scan was used to observe changes in the bone structure of the hind paws of mice in the sixth week. (**E**) Micro-CT scanning quantitative analysis. BV/TV, bone volume/tissue volume. All data compared with the normal group showed * *p* < 0.05, *** *p* < 0.001; those compared with the model group showed ## *p* < 0.01.

Recent studies have confirmed that APS exerts metabolic regulatory and anti-inflammatory effects in diabetes and infectious diseases. APS has also been shown to alleviate RA symptoms; however, the underlying mechanisms remain elusive. In the present study, APS was administered to CIA mice for both prophylactic and therapeutic purposes. Following CIA induction, the mice developed pathological features typical of RA, including marked swelling and deformity of the hind paw joints. Notably, these symptoms were significantly ameliorated after APS prophylactic or therapeutic intervention (Figure 1). Compared with the methotrexate-treated model group, the number of arthritic mice in both the APS prophylactic and therapeutic groups was reduced by day 60 (Figure 2A). The histopathological scores in these two groups began to outperform those of the model group from the second week of treatment (Figure 2B). Except for the normal control group, the body weight of mice in all other groups exhibited a trend consistent with that in the CIA group, indicating that the tested drugs at the selected doses exerted no potential toxic effects on mice (Figure 2C). Micro-CT analysis revealed severe articular surface destruction accompanied by osteophyte formation in the hind paws of the model group mice. Following APS prophylaxis or therapy, structural joint damage was mitigated, and osteophyte formation decreased (Figure 2D). And the weight Further bone volume/total volume (BV/TV) analysis demonstrated that the BV/TV values of both APS-treated groups were significantly superior to those of the model group, with the therapeutic group exhibiting even better outcomes (Figure 2E). Collectively, these results confirm that APS effectively suppresses joint damage and pathological progression, thereby alleviating the course of RA.

### 2.2. APS Restores Th17/Treg Balance in RA Mice

**Figure 3:**
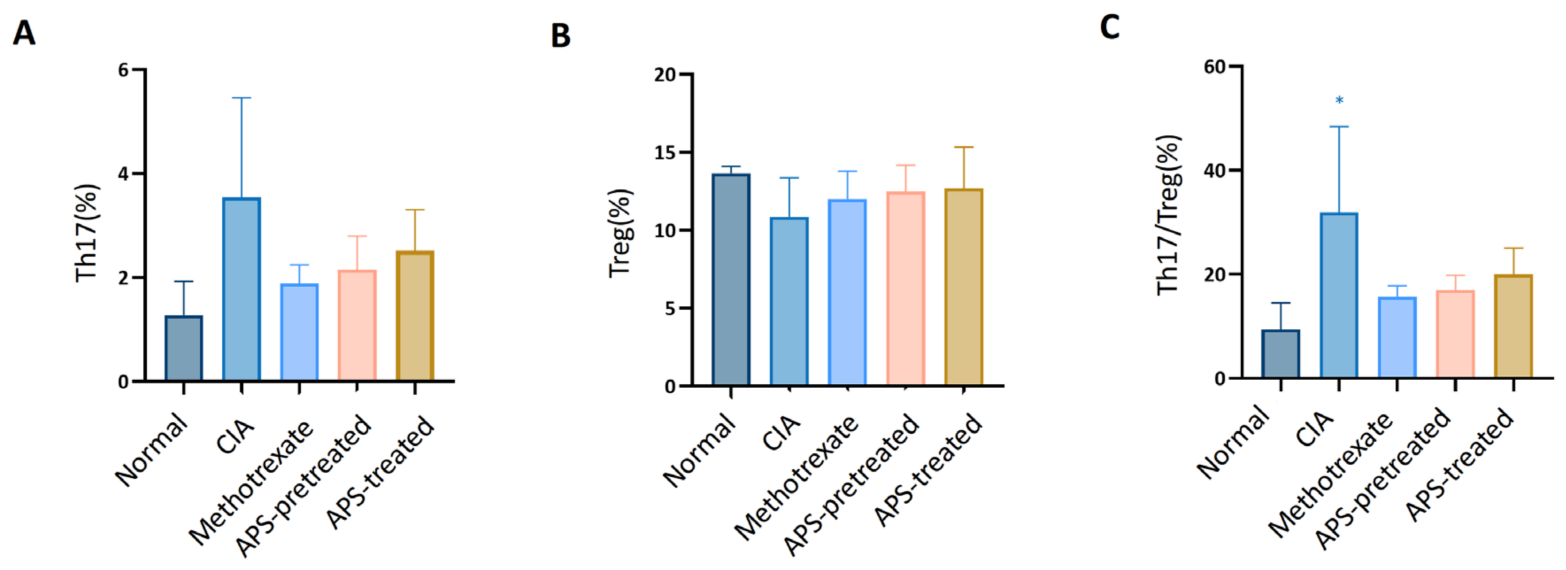
Detection of Th17 and Treg cell frequencies in mouse spleen. Spleen lymphocytes were obtained from the normal, CIA, CIA + methotrexate, APS-pretreated, and APS-treated groups of mice and measured via flow cytometry. (**A**) The frequency of Th17 cells. Th17: CD4^+^IL-17^+^ T cells. (**B**) The frequency of Treg cells. Treg: CD4^+^Foxp3^+^ T cells. (**C**) The ratio of Th17 to Treg cells (*n* = 3). All data compared with the normal group showed * *p* < 0.05.

APS has been reported to maintain Th17/Treg homeostasis by modulating gut microbiota composition; however, its role in RA remains poorly characterized. Spleen lymphocytes were isolated, and the frequencies of Th17 and Treg cells were determined via flow cytometry. Compared with the model group, the frequency of Th17 cells was significantly decreased in the spleens of both the APS prophylactic and therapeutic groups (Figure 3A), whereas the frequency of Treg cells was moderately increased (Figure 3B), resulting in a reduced Th17/Treg ratio (Figure 3C).

**Figure 4:**
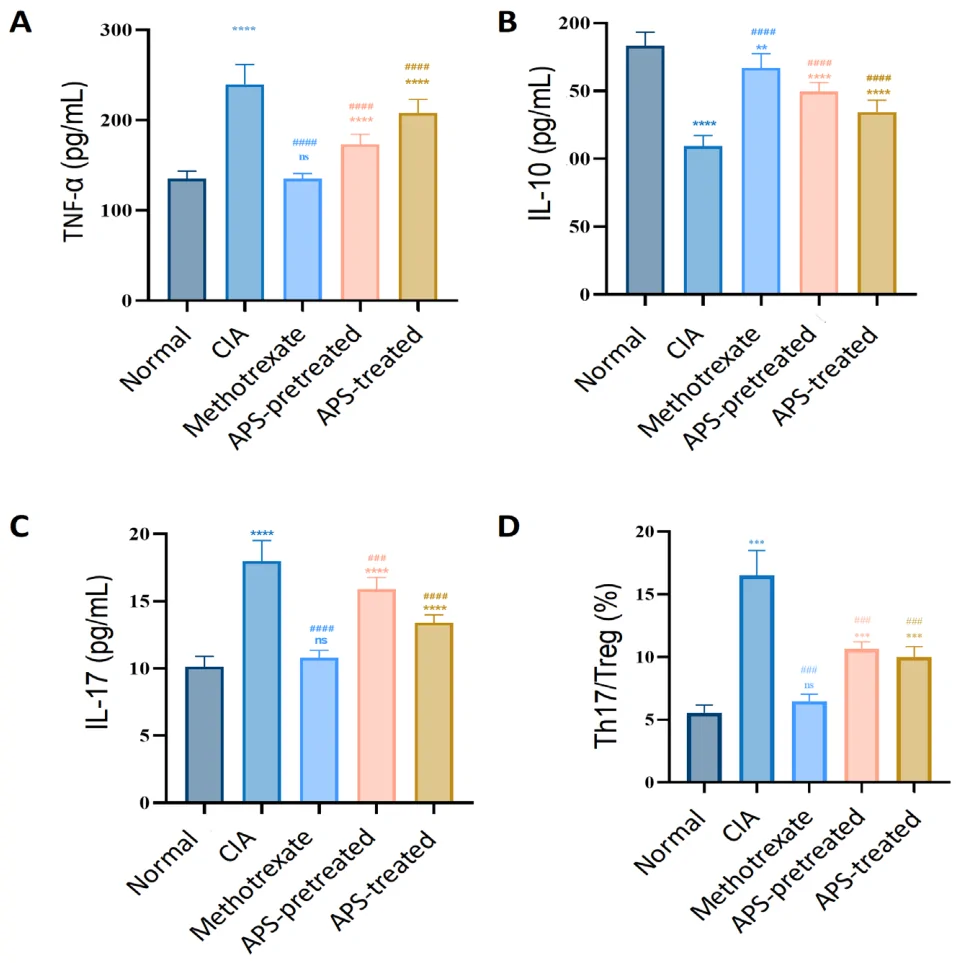
Detection of inflammatory cytokine concentrations in mouse serum. The concentrations of inflammatory cytokines were detected in the serum of mice in the normal, CIA, CIA + methotrexate, APS-pretreated, and APS-treated groups in week 6 using ELISA. (**A**) TNF-α, (**B**) IL-10, (**C**) IL-17, (**D**) Th17/Treg ratio. All data compared with the normal group showed ** *p* < 0.01, *** *p* < 0.001 or **** *p* < 0.0001; those compared with the model group showed ### *p* < 0.001 or #### *p* < 0.0001.

Furthermore, serum concentrations of TNF-α, IL-10 (Treg), and IL-17 (Th17) were measured via ELISA. The results demonstrated that, compared with the model group, serum TNF-α levels were significantly reduced in both the APS prophylactic and therapeutic groups, with the prophylactic group showing superior efficacy (Figure 4A). IL-10 concentrations showed a similar trend, with more pronounced improvements observed in the prophylactic group (Figure 4B). IL-17 concentrations reflect the activation status of Th17 cells in vivo. Compared with the model group, IL-17 levels were significantly decreased in both APS-treated groups; however, the therapeutic group outperformed the prophylactic group in this regard (Figure 4C), suggesting that APS may exert a more direct regulatory effect on Th17 cells. Analysis of the IL-17 (Th17)/IL-10 (Treg) ratio revealed that the Th17/Treg balance shifted toward a healthy state following APS intervention (Figure 4D).

These results confirm that APS effectively improves helper T cell homeostasis in vivo, restoring the disrupted Th17/Treg balance toward normality, thereby contributing to the amelioration of RA symptoms.

### 2.3. APS Improves Gut Microbiota Diversity and Composition in CIA Mice

Intestinal microbial dysbiosis and immune dysregulation represent critical triggers in the pathogenesis of RA [14]. APS has been demonstrated to improve gut microbiota composition, reshape host Th17/Treg cell balance, and alleviate symptoms of ulcerative colitis and experimental autoimmune encephalomyelitis [15,16].

In the present study, CIA was established in mice, which were subsequently administered APS for 6 weeks. Fecal samples were collected from these donor mice and transplanted into recipient CIA mice. Fecal specimens were obtained from the recipients six weeks post-transplantation. 16S rRNA sequencing was performed on fecal samples from the normal control, CIA, and fecal microbiota transplantation (FMT) recipient (CIA + APS-gut microbiota) mice to assess species richness and evenness.

**Figure 5:**
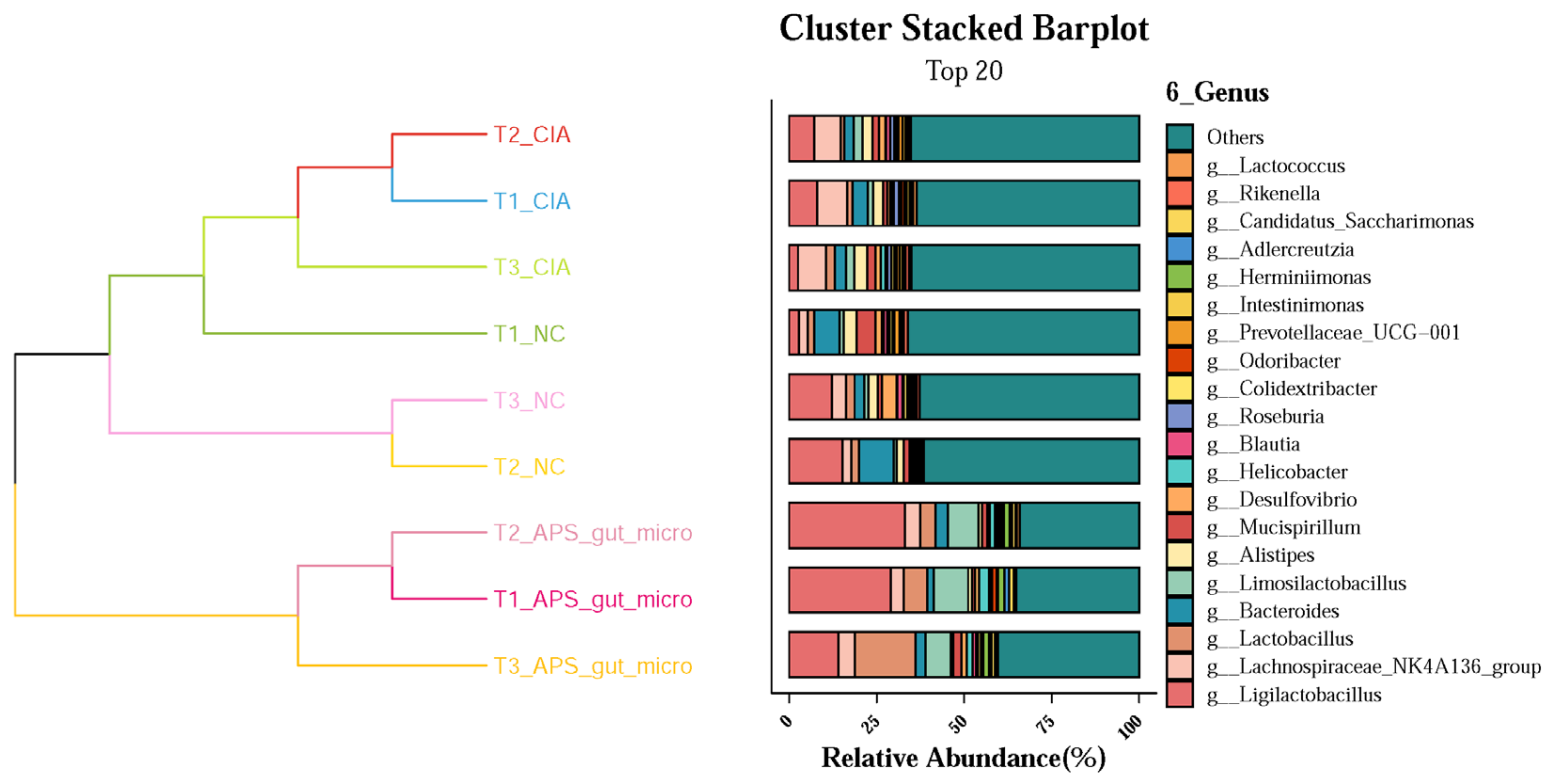
16S rRNA gene sequencing-based analysis of the gut microbiome. Feces were collected from the normal, CIA, and CIA + APS gut microbiota mice at different time points (T1, T2, and T3 represent 10, 20, and 30 days after gavage, respectively) for microbial 16S rRNA sequencing. Genus-level relative abundance results are presented in a clustered stacked bar chart, enabling comparison of the microbial composition between the groups.

Community clustering analysis based on species richness revealed that the genus Rikenella was markedly depleted in the fecal microbiota of CIA mice, whereas its abundance was significantly restored following FMT, indicating enhanced antioxidant and anti-inflammatory capacities in the host. Furthermore, FMT resulted in a notable enrichment of beneficial bacterial genera, including Ligilactobacillus, Lactobacillus, Limosilactobacillus, and Bacteroides, corroborating the enhancement of host anti-inflammatory and immunomodulatory functions (Figure 5).

Collectively, these findings demonstrate that transplantation of fecal microbiota from APS-treated mice into CIA recipients remodeled the intestinal microbial community. Exogenous probiotic organisms established colonization through competitive exclusion, thereby suppressing pathogenic taxa and substantially increasing the relative abundance of beneficial genera such as Ligilactobacillus, ultimately augmenting the intrinsic anti-inflammatory capacity of the host.

**Figure 6:**
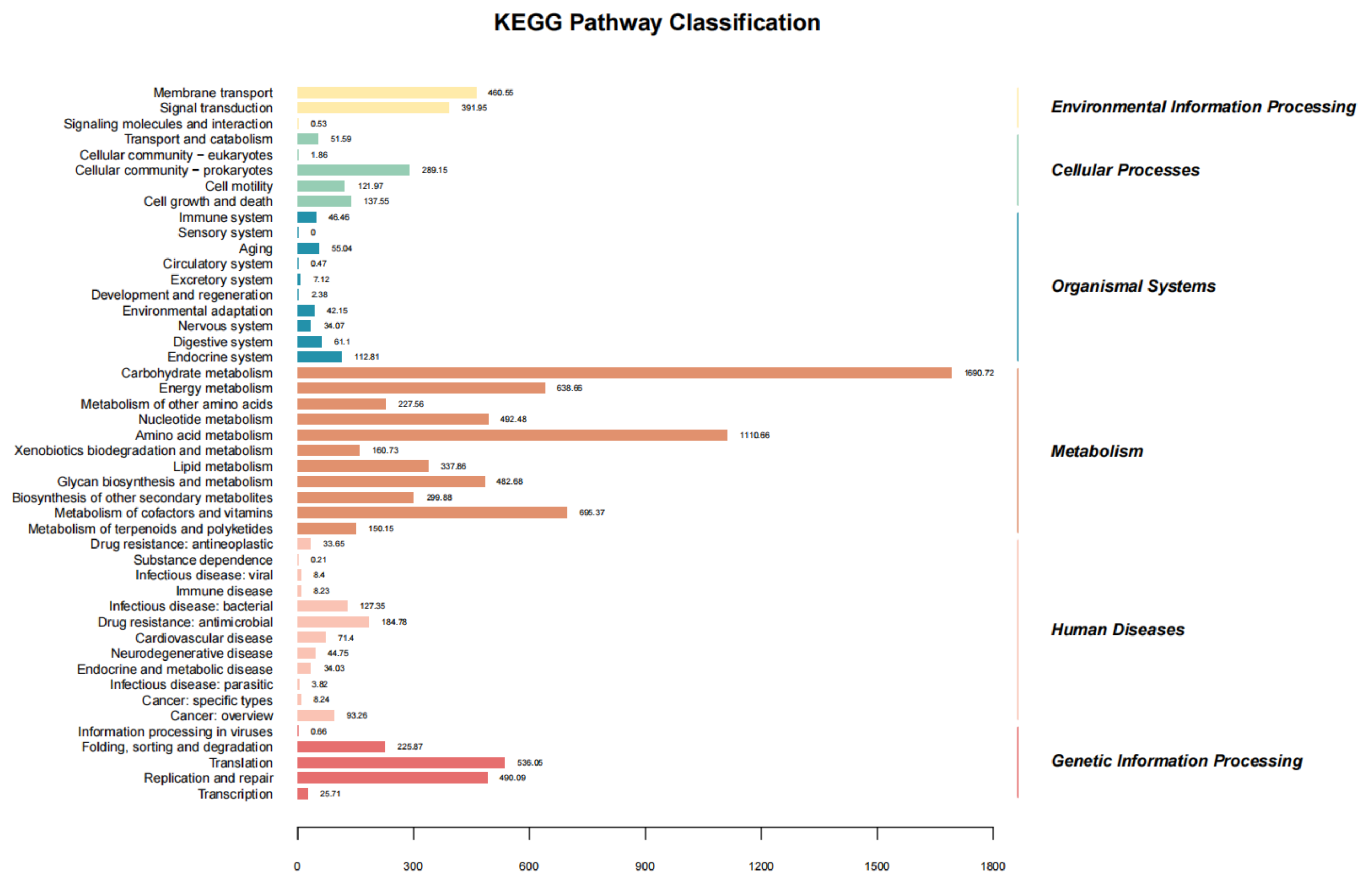
Analysis of predicted metabolic pathways of gut microbiota in mice. Differential functional abundance is presented in an extended error bar plot.

Predictive functional profiling of microbial genes revealed elevated activity in pathways associated with metabolism, genetic information processing, and environmental information processing, suggesting that APS modulates host pathophysiological processes through the regulation of these microbial signaling pathways (Figure 6).

### 2.4. APS Alleviates RA Symptoms by Improving Gut Microbiota Composition

**Figure 7:**
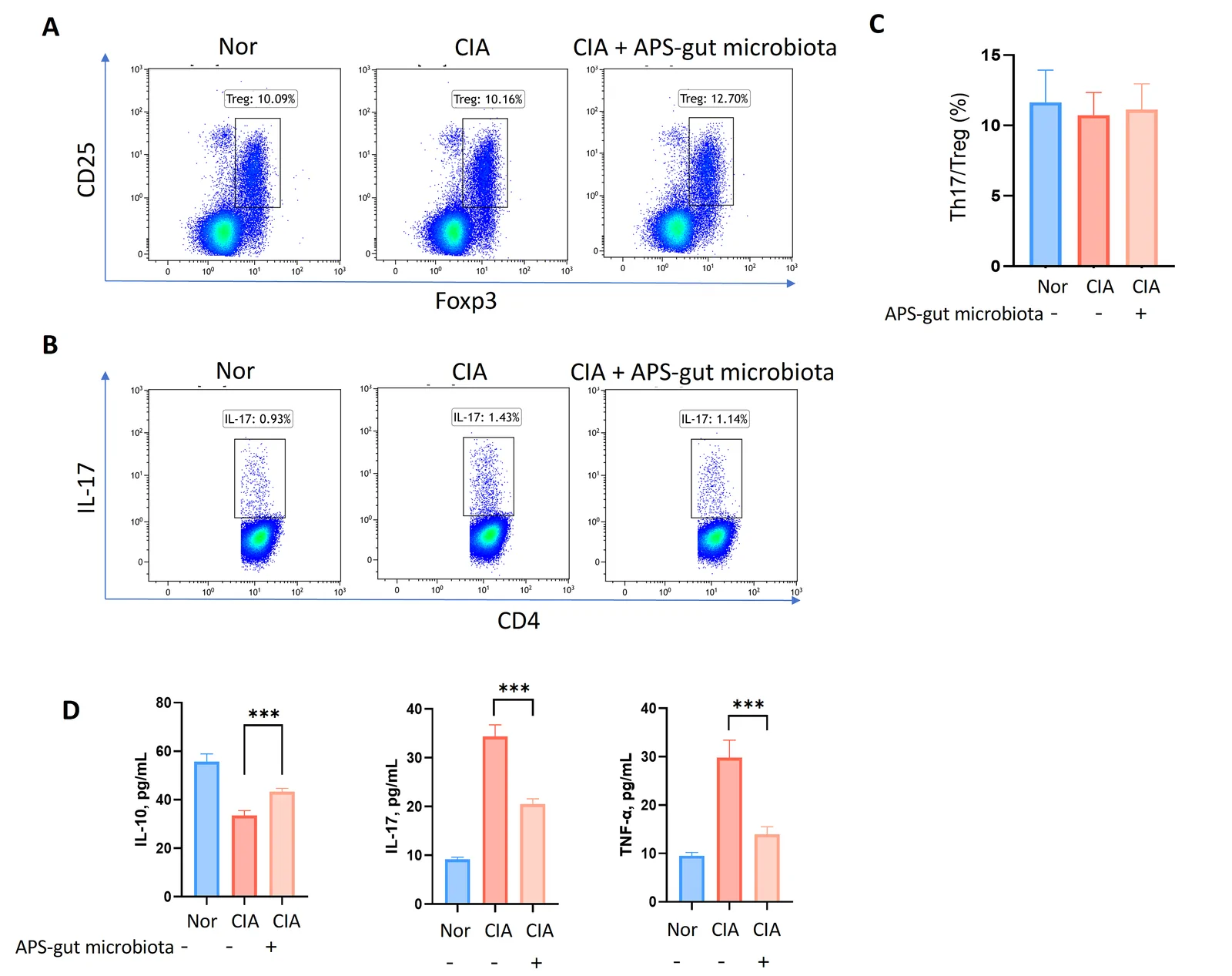
Frequencies of Th17 and Treg cells in the spleen and concentrations of inflammatory mediators in serum after FMT. Spleen lymphocytes were obtained from normal, CIA, and CIA + APS gut microbiota group mice and measured via flow cytometry. (**A**) The frequency of Treg cells. Treg: CD4^+^Foxp3^+^ T cells. (**B**) The frequency of Th17 cells. Th17: CD4^+^IL-17^+^ T cells. (**C**) The ratio of Th17 to Treg cells (*n* = 3). CIA + APS gut microbiota: CIA (FMT recipient) refers to RA mice receiving FMT, and APS gut microbiota (FMT donor) refers to the fecal microbiota of RA mice after 6 weeks of APS treatment. (**C**) The ratio of Th17 to Treg cell frequency (*n* = 3). (**D**) Concentrations of IL-10, IL-17A and TNF-α in serum. All data compared with the normal group showed *** *p* < 0.001.

Gut microbiota alterations can directly affect host immune homeostasis, particularly the Th17/Treg balance [17,18]. Gut-resident *Lactobacillus* modulates the Th17/Treg balance, thereby stabilizing immune homeostasis and alleviating the pathological manifestations of inflammatory bowel disease (IBD) [19]. APS has also been demonstrated to ameliorate disrupted intestinal homeostasis and correct the Th17/Treg imbalance [15]. Splenic lymphocytes were isolated from normal, CIA, and CIA + APS-FMT mice, and the frequencies of Treg (CD4^+^Foxp3^+^ T) and Th17 (CD4^+^IL-17^+^ T) cells were analyzed via flow cytometry. The results showed that the frequency of both splenic Treg cells (Figure 7A) and Th17 cells (Figure 7B,C) increased following fecal transplantation. The mouse serum concentrations of IL-17, IL-10 and TNF-α were also measured before and after FMT. It was found that the concentrations of pro-inflammatory factors such as IL-17 and TNF-α significantly decreased after FMT, while the anti-inflammatory factor IL-10 showed the opposite trend (Figure 7D). Thus, the results indicate that inflammation was alleviated in the APS-treated mice.

**Figure 8:**
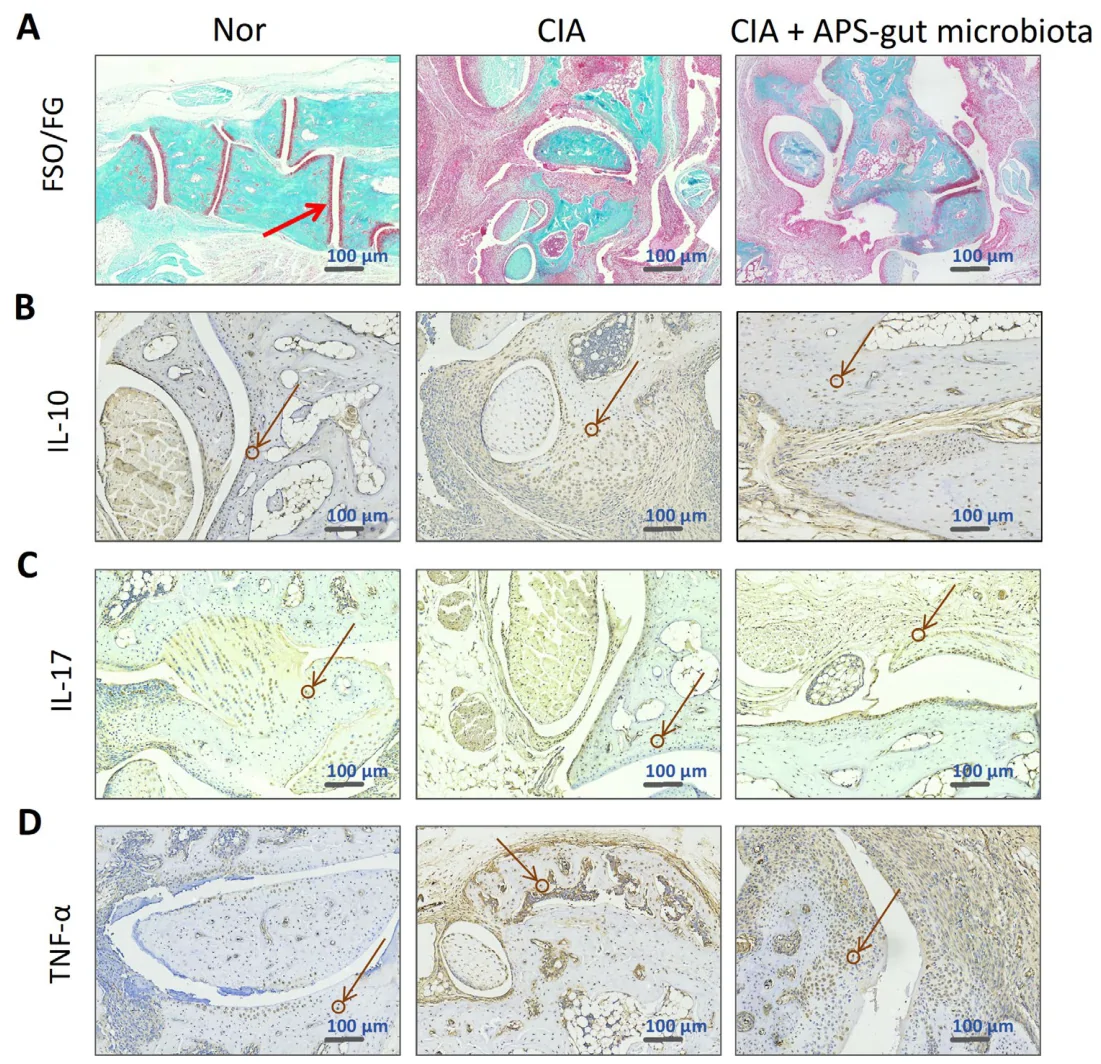
Histopathological examination of mice articular tissues. Mice in the normal, CIA, and CIA + APS gut microbiota groups were dissected, and hind paw articular tissues were isolated. (**A**) SO/FG. Immunohistochemical detection for analysis of IL-10 (**B**), IL-17 (**C**), and TNF-α (**D**) protein levels. The red arrows indicate the staining of cartilage, while the brown arrows and circles indicate representative regions of positive immunostaining for IL-10, IL-17 and TNF-α, respectively; brown chromogenic signals denote positive protein expression. Scale bar, 100 μm.

The mice were euthanized, and articular tissues were isolated, embedded, sectioned, and stained with Safranin O–fast green to evaluate bone erosion. The results showed that, following CIA induction, both cartilage and bone tissues were severely eroded, with blurred cartilage interfaces. After APS treatment, bone destruction was attenuated, and cartilage interfaces appeared to be more intact (Figure 8A). Immunohistochemical analysis revealed that the anti-inflammatory cytokine IL-10 was downregulated in CIA mice but upregulated following fecal transplantation (Figure 8B). In contrast, the pro-inflammatory cytokines IL-17 and TNF-α showed opposite trends (Figure 8C,D), suggesting that APS modulates gut microbiota and thus influences host immune homeostasis, thereby suppressing pro-inflammatory immune cell infiltration, reducing inflammatory cytokine accumulation in joint tissues, and consequently delaying joint pathological progression.

## 3. Discussion

Although biologics have been widely applied in clinical practice for RA treatment, a substantial proportion of patients fail to respond or experience secondary loss of efficacy, with some exhibiting suboptimal radiographic progression [20]. Therefore, there is an urgent need to identify novel therapeutics that can address these clinical challenges. Plant-derived compounds, as natural products with favorable safety profiles, have been extensively investigated in experimental and preclinical studies of RA [21,22,23]. APS has also been demonstrated to delay RA pathogenesis [9,10]; however, the specific mechanisms require further elucidation. In this study, we demonstrated that APS alleviates CIA in mice by optimizing gut microbiota composition, restoring the Th17/Treg balance, reducing inflammatory cell infiltration in joint tissues, suppressing systemic pro-inflammatory responses, and mitigating joint bone damage.

Synovial hyperplasia and inflammatory lesions in affected joints are the main pathological features of RA [9]. Our findings revealed that APS effectively suppresses bone tissue erosion and alleviates joint pathological changes in CIA mice, regardless of whether administered prophylactically or therapeutically. APS modulates inflammation through regulation of the Th17/Treg balance and restoration of immune homeostasis [15]. Our study also confirmed that APS effectively improves helper T cell homeostasis in vivo. In APS-treated mice, splenic Th17 cell frequencies significantly decreased, whereas Treg cell frequencies increased, restoring the disrupted Th17/Treg balance toward normality. Concurrently, serum concentrations of the pro-inflammatory cytokines TNF-α and IL-17 were significantly reduced following APS prophylaxis or therapy, while anti-inflammatory cytokine IL-10 levels were correspondingly elevated. These changes collectively contribute to the amelioration of RA symptoms.

The gut–immune axis represents a critical node in the pathogenesis of RA. Intestinal microorganisms participate in host signal transduction and immune regulation through the release of metabolites, thereby triggering RA pathogenic mechanisms. APS has been shown to improve gut microbiota composition, reshape host Th17/Treg cell balance, and alleviate symptoms of ulcerative colitis and experimental autoimmune encephalomyelitis [15,16]. We found that APS significantly increased the proportions of the lactic acid bacteria *Ligilactobacillus*, *Lactobacillus*, *Limosilactobacillus*, and *Bacteroides* in the intestines of CIA mice, confirming enhanced immunomodulatory capacity and intestinal microenvironment remodeling. APS reshapes the gut microbiota, which subsequently modulates host pathways governing metabolism, genetic information processing, and environmental information processing, ultimately ameliorating the pathological phenotype. These substances may participate as microbial and host metabolic byproducts. Gut microbiota alterations can influence the Th17/Treg balance [17,18]; among the microbiota, *Lactobacillus* serves as a pivotal beneficial regulator [19], and APS has been demonstrated to ameliorate disrupted intestinal homeostasis and correct the Th17/Treg imbalance [15]. Our study further confirmed that APS increases intestinal *Ligilactobacillus* abundance and suppresses harmful bacteria, thereby enhancing Treg cell frequencies in CIA mice and restoring the Th17/Treg balance. As demonstrated in studies [24,25], the Th17/Treg immune axis governs autoimmune inflammation in RA/CIA, Th17 and Treg are reciprocally regulated populations, and small proportional shifts in both subsets may collectively alter the Th17/Treg ratio and reshape immune homeostasis. Consistent with our observation, composite improvement of the Th17/Treg ratio can accompany ameliorated arthritic phenotype, even when the individual subset changes are mild and lack statistical significance. Inflammatory mediators in the body also showed a trend of moving toward healthy levels, with reduced inflammatory cell infiltration in affected joints.

In conclusion, we demonstrated that APS is a safe and effective natural compound suitable for preventing and treating RA. It enhances the abundance of intestinal probiotics, suppresses harmful bacteria, remodels the disrupted gut environment in RA, restores the Th17/Treg balance, and corrects dysregulated inflammatory states. Locally, in affected joints, APS suppresses pro-inflammatory immune cell infiltration, restrains pro-inflammatory mediator release and accumulation, reduces inflammatory responses at joint sites, and delays disease progression.

## 4. Materials and Methods

### 4.1. Reagents

The following reagents were used in this study: astragalus polysaccharide (SA9790, Solarbio, Beijing, China), Complete Freund’s Adjuvant (7001, Chondrex, Woodinville, WA, USA), Incomplete Freund’s Adjuvant (7022, Chondrex, Woodinville, WA, USA), immunization-grade bovine type II collagen solution (20022, Chondrex, Woodinville, WA, USA), methotrexate (IM0160, Solarbio, Beijing, China), 4% paraformaldehyde (PFA) fixative (G1101), Ethylenediaminetetraacetic Acid (EDTA, GC202001), 4,6-Diamidino-2-phenylindole (DAPI, G1012) (Servicebio, Wuhan, China), mouse lymphocyte separation solution (7211011, Dakewe, Shenzhen, China), Cell Activation Cocktail (without Brefeldin A) (4223302, Biolegend, San Diego, CA, USA), Cyto-Fast Fix/Perm Buffer Set (426803, Biolegend, San Diego, CA, USA), FITC anti-mouse CD4 (100510, Biolegend, San Diego, CA, USA), APC anti-mouse CD25 (1020100, Biolegend, San Diego, CA, USA), Foxp3 Monoclonal Antibody (FJK-16S), PE-Cyanine7 (25-5773-80, Invitrogen, Carlsbad, CA, USA), Cell Staining Buffer (420201, Biolegend, San Diego, CA, USA), TruStain FcX (anti-mouse CD16/32) (101319, Biolegend, San Diego, CA, USA), True-Nuclear Transcription Factor Buffer Set (424401, Biolegend, San Diego, CA, USA), Zombie Yellow™ Fixable Viability Kit (423103, Biolegend, San Diego, CA, USA), Mouse Tumor Necrosis Factor α (TNF-α) ELISA Kit (ED-20852, Xiamen LunChangShuo Biotechnology Co., Ltd. Xiamen, China), Mouse Interleukin 10 (IL-10) ELISA Kit (ED-20162, Xiamen LunChangShuo Biotechnology Co., Ltd. Xiamen, China), Mouse Interleukin 17 (IL-17) ELISA Kit (ED-2170, Xiamen LunChangShuo Biotechnology Co., Ltd. Xiamen, China), IL-10 Polyclonal Antibody (208502-1-AP, Proteintech, Wuhan, China), IL-17 Polyclonal Antibody (13082-1-AP, Proteintech, Wuhan, China), TNF-Alpha Polyclonal Antibody (137590-1-AP, Proteintech, Wuhan, China), and E.Z.N.A.^®^ Mag-Bind^®^ Stool DNA Kit (M4015-02, Omega Bio-tek. Norcross, GA, USA).

### 4.2. Animals

In this study, male DBA/1 mice, suitable for rheumatoid arthritis models, were used. The mice (6–8 weeks) were acclimatized for 1 week prior to induction of collagen-induced arthritis (CIA). Equal volumes of Complete Freund’s Adjuvant (CFA) and immunization-grade bovine type II collagen solution were mixed and emulsified on ice using a high-speed homogenizer. The emulsion (0.1 mL, containing 100 μg collagen) was injected intradermally at 2 distinct sites in the gluteal region. The day of primary immunization was designated as day 1. A booster immunization was administered on day 21 using an identical dose and procedure.

Oral doses of APS (100–300 mg/kg/day) have been reported to be effective and well tolerated in mouse models of arthritis [26,27]. Based on previous studies, the dosage of APS used in our experiment was 100 mg/kg/day. Accordingly, the mice were randomly allocated to five groups (*n* = 8 per group): a normal (healthy control) group, a CIA group, a positive control group (methotrexate, 0.065 mg/kg), an astragalus polysaccharide pre-treatment group (APS-pretreated, 100 mg/kg), and an astragalus polysaccharide treatment group (APS-treated, 100 mg/kg). The mice in the normal and CIA groups received phosphate-buffered saline (PBS) daily via gavage; those in the APS-pretreated and APS-treated groups were dosed with drug suspensions via gavage daily for 42 days; and those in the methotrexate group received drug suspensions via gavage once weekly.

After CIA induction, the mice were randomly assigned to treatment groups using a random number table, stratified by baseline arthritis score to ensure comparable disease severity among the groups at baseline.

### 4.3. Assessment of Joint Swelling

The progression of joint morphology was monitored and recorded. Arthritis severity was scored independently by two investigators, blinded to group allocation, according to the criteria described by Brand et al. [28]. Joint scores were recorded weekly; the maximum possible score was 16 per mouse.

### 4.4. Micro-CT

After 42 days of drug administration, the mice were anesthetized via an intraperitoneal injection of 5% chloral hydrate and humanely killed via cervical dislocation. The hind paws were rapidly excised, and the surrounding skin, fur, and excess soft tissue were carefully removed. Specimens were fixed in 4% neutral paraformaldehyde for 24 h. VivaCT 40 In Vivo micro-computed tomography (Micro-CT) (SCANCO Medical AG, Switzerland) was employed to evaluate morphological alterations in the hind paw bones. The scanning parameters were as follows: source voltage, 70 kV; source current, 114 μA; and spatial resolution, 20 μm. The acquired raw tomographic image data were imported into Mimics software 25.0 (Materialise NV, Leuven, Belgium) in DICOM format. Standard bone tissue gray value thresholds were applied for region segmentation to eliminate residual soft tissue noise, and any discrepancies in region-of-interest (ROI) delineation were resolved by consensus between two independent observers. Three-dimensional reconstruction was subsequently performed to generate high-precision, complete three-dimensional anatomical images of the hind paw bones for subsequent qualitative and quantitative morphological assessments.

### 4.5. Flow Cytometry

The spleens were excised and processed into single-cell suspensions. Lymphocytes were isolated using mouse lymphocyte separation solution. The cells were subjected to live/dead staining, Fc receptor blocking, and fluorescent antibody labeling, followed by incubation at 4 °C in the dark for 30 min. Fixation was performed using 4% paraformaldehyde. Flow cytometry was used to determine the frequencies of Th17 (CD4^+^IL-17^+^) and Treg (CD4^+^CD25^+^Foxp3^+^) cells.

### 4.6. Enzyme-Linked Immunosorbent Assay (ELISA)

Blood samples were collected and centrifuged at 3000 rpm for 10 min. Serum supernatants were harvested and stored at −80 °C. Serum concentrations of TNF-α, IL-10, and IL-17 were determined in strict accordance with the manufacturer’s protocols.

### 4.7. Fecal Microbiota Transplantation (FMT)

CIA was induced in mice as described in Section 2.2. The mice were then randomly divided into three groups: normal, CIA, and CIA + APS-gut microbiota groups (*n* = 5 per group). Fecal samples from the RA treatment group (APS-pretreated group) were collected and resuspended in 1 mL of sterile water. The suspension was centrifuged at 800× *g* for 3 min, and the resulting supernatant was reserved. Beginning on the day after the second immunization, the supernatant was administered to the mice in the CIA + APS-gut microbiota group via gavage as fecal microbiota transplantation three times per week for 4 weeks at a dose of 100 μL per mouse.

### 4.8. 16S rRNA Sequencing of Gut Microbiota

Following gavage, fecal samples (approximately 0.2–0.5 g) were collected from each group on days 10 (T1), 20 (T2), and 30 (T3). Total DNA was extracted using the E.Z.N.A.^®^ Stool DNA Kit (M4015-02, Omega Bio-tek, Norcross, GA, USA) according to the manufacturer’s protocol. DNA concentration and purity were assessed using a NanoDrop 2000 spectrophotometer (Thermo Fisher Scientific, Waltham, MA, USA. OD_260_/OD_280_ = 1.8–2.0), and integrity was verified via 1% agarose gel electrophoresis. The samples were stored at −20 °C until further analysis. 16S rRNA sequencing was performed by Hangzhou Lianchuan Biotechnology Co., Ltd. (Hangzhou, China) Species richness and community clustering were analyzed. Microbial abundance was compared amongst the normal, CIA, and CIA + APS-gut microbiota groups, and potential functional pathways were predicted.

### 4.9. Safranin O–Fast Green Staining (SO/FG)

Following humane killing, the right hind limbs were removed. Skin and muscle were carefully dissected to preserve the integrity of the knee joint. The hind paws were fixed in 4% paraformaldehyde for 24 h and subsequently decalcified in 10% EDTA solution for 21 days. The specimens were then embedded, sectioned, and stained with Safranin O–fast green. Cartilage damage was examined via microscopy, and the severity was assessed by the same two independent blinded observers.

### 4.10. Determination of Serum Inflammatory Factors

Serum collection from FMT groups was performed at the end of the experimental period (T3). Briefly, blood samples were collected from the orbital venous plexus of anesthetized mice and centrifuged at 3000 rpm for 10 min. Serum supernatants were harvested and stored at −80 °C. Serum concentrations of TNF-α, IL-10, and IL-17 were determined in strict accordance with the manufacturer’s protocols by ELISA.

### 4.11. Immunohistochemistry

Tissue sections were dewaxed, rehydrated, permeabilized, and blocked with serum. IL-10 (1:200), IL-17 (1:200), and TNF-α (1:200) primary antibodies were applied and incubated overnight at 4 °C. After three washes with PBS, biotin-conjugated goat anti-rabbit secondary antibodies (1:200) were added and incubated at room temperature for 1 h. The sections were washed 3 times with PBS, followed by the addition of reagent D. DAB chromogenic substrate was then applied for visualization. The sections were subsequently dehydrated, cleared, and mounted. Negative control sections were prepared with isotype-matched IgG instead of primary antibody under identical conditions to exclude non-specific background staining; these controls were used for staining threshold assessment. Images were captured using an upright light microscope, and the extent of cellular staining was assessed independently by two blinded observers. The immunoreactive score was adopted, calculated as the product of staining intensity and the percentage of positive cells. Any scoring discrepancies were resolved through consensus discussion.

### 4.12. Statistical Analysis

All data were analyzed using GraphPad Prism 10 software. Student’s *t*-test was used for comparisons between two groups, and one-way ANOVA, followed by Tukey’s test, was used for comparisons among multiple groups. All data are expressed as the mean ± standard deviation. Statistical significance was set at *p* < 0.05.

## 5. Study Limitations

In the CIA model, female DBA/1 mice are responsive to estrogen and relatively resistant to disease induction, whereas male mice exhibit more stable disease progression. To ensure a satisfactory model success rate, male mice were therefore selected based on the reagent instructions and published literature on the CIA model. This study did not include in-depth profiling of microbial metabolites and was limited to preliminary functional prediction based on 16S rRNA sequencing data, which precluded a deeper analysis of the molecular mechanisms underlying microbiota–immune interactions. Although the effect size was large and consistent with other readouts, the small sample size necessitates validation in larger-scale studies. In addition, pharmacokinetic analyses of APS absorption and biodistribution were not performed, and these will be addressed in future studies. These limitations highlight directions for future research and emphasize that the main contribution of this study lies in the initial establishment of a causal association framework for the APS–microbiota–Th17/Treg axis.

## Data Availability

The original contributions presented in this study are included in the article. Further inquiries can be directed to the corresponding author.
